# Strengthening Community Health Systems Through Novel eHealth Initiatives? Commencing a Realist Study of the Virtual Health Rooms in Rural Northern Sweden

**DOI:** 10.34172/ijhpm.2021.08

**Published:** 2021-02-09

**Authors:** Frida Jonsson, Dean B. Carson, Isabel Goicolea, Anna-Karin Hurtig

**Affiliations:** ^1^Department of Epidemiology and Global Health, Umeå University, Umeå, Sweden.; ^2^Arctic Research Centre (Arcum), Umeå University, Umeå, Sweden.; ^3^School of Business and Law, CQUniversity, Rockhampton, QLD, Australia.

**Keywords:** Northern Sweden, eHealth, Realist Evaluation, Community Health System, Person-Centred Care

## Abstract

**Background:** Unlike the large body of research that has examined the ‘success’ or ‘failure’ of eHealth in terms of patient and provider perceptions or cost- and clinical effectiveness, the current study teases out ways through which a novel eHealth initiative in rural northern Sweden might result in more distal or systemic beneficial outcomes. More specifically, this paper aims to explore how and under what circumstances the so-called virtual health rooms (VHRs) are expected to improve access to person-centred care and strengthen community health systems, especially for elderly residents of rural areas.

**Methods:** The first phase of the realist evaluation methodology was conducted, involving qualitative interviews with 8 key stakeholders working with eHealth, business development, digitalisation, and process management. Using thematic analysis and following an abductive-retroductive analytical process, an intervention-context-actor-mechanism-outcome(ICAMO) configuration was developed and elicited into an initial programme theory.

**Results:** The findings indicate that a novel eHealth initiative, which provides reliable technologies in a customized facility that connects communities and providers, might improve access to person-centred care and strengthen community health systems for rural populations. This is theorized to occur if mechanisms acting at individual (such as knowledge, skills and trust) and collective (like a common vision and shared responsibilities) levels are triggered in contexts characterised by supportive societal transitions, sufficient organisational readiness and the harnessing of rural cohesiveness and creativity.

**Conclusion:** The elicited initial programme theory describes and explains how a novel eHealth initiative in rural northern Sweden is presumed to operate and under what circumstances. Further testing, refinements and continued gradual building of theory following the realist evaluation methodology is now needed to ascertain if the ‘VHRs’ work as intended, for whom, in what conditions and why.

## Background

**Key Messages**

** Implications for policy makers**

- Substantial distal and systemic beneficial outcomes following from the implementation of novel eHealth initiatives can be achieved in sparsely populated and remote areas if health services are empowered to drive a shared vision and establish locally relevant goals.
- Many eHealth initiatives are driven by enthusiasm for the technology, but more attention needs to be paid to ensuring that there is time and knowledge devoted the process of implementation which usually extends well beyond the initial ‘project’ phase.
- While specific eHealth initiatives usually rely on the engagement of individuals or small groups, successful implementation requires well-articulated responsibilities, coordination and communication strategies across all stakeholders.
- eHealth initiatives, which organises healthcare in new ways while providing reliable technologies in customized facilities that connects rural populations and distant providers, have the potential to strengthen community health systems and improve access to person-centred care.

** Implications for the public**

eHealth offers tremendous potential for improving the provision and delivery of healthcare services in isolated rural communities. However, despite research generally indicating clinical effectiveness, initiatives that use information and communication technologies for health has so far been scarcely documented in real-world settings and largely implemented in a trial-and-error fashion, with little theoretical basis to support them. To address these knowledge gaps, our research exploring ideas and expectations that underlie the design and development of the so-called virtual health rooms (VHRs) in rural northern Sweden, highlight the importance of considering how and under what circumstances eHealth initiatives are introduced and implemented. In particular, this study suggests that initiatives in eHealth – like the VHRs – might improve access to person-centred care and strengthen community health systems for rural populations if they engender trust and cooperation among actors who have a common vision while sharing responsibilities and resources in mutual dependence.

Ageing populations with complex health needs have shifted thinking away from hospital-centric and curative approaches towards flexible and personalized models of care, particularly in high-income countries.^1^ This change has contributed to a rising interest in and concern about the organisation of primary healthcare (PHC) in Sweden more generally, and rural areas in particular, with the system largely struggling in the role of ‘front-line care’ and to manage the increased multi-morbidities of a growing elderly population.^2^ Based partly on these problems, a large reform has recently been introduced to develop an equitable, accessible and efficient Swedish health system. In particular, this policy has been focused on strengthening PHC, improving the coordination between provinces and municipalities, promoting access to person-centred solutions and facilitating the integration of medical technologies.^3^ However, while initiatives that use information and communication technologies for health are assumed to make clinical support structures more effective, improve patient-provider connection and increase peoples’ involvement in their care processes, eHealth has yet to show its expected potential in terms of scale, full system deployment and/or effectiveness for health services, especially at the community level.^4^

The body of research studying factors that contribute to the ‘success’ or ‘failure’ of eHealth has grown rapidly since the mid- 1990s. To date, the field comprises a multitude of reviews^5-8^ (and even reviews of reviews^9,10^) focusing on aspects such as financial costs and benefits, patient and provider perceptions, and clinical effectiveness; also in specific domains relevant for this study such as elderly care,^11,12^ rural/remote health^13,14^ and rural elderly care.^15,16^ Since the extent to which eHealth initiatives work or not is contingent upon appropriate design, implementation *and *utilization, scholars have identified a number of central aspects in these processes. Previous studies have, for example, highlighted the importance of users being ‘digitally literate’^17^; of creating ‘real’ engagement and collaboration between stakeholders^18^; and of ensuring that the interventions become part of formal systems and routine care.^19^

Studies to date have been largely concerned with whether individual eHealth applications are used as intended, either for the desired clinical outcomes or with regard specific populations^14^ thus focusing on the intervention’s ‘hard core’ (eg, tools and referral systems) stripped of its broader context and ‘soft periphery’ (eg, values and relationships). However, since individual projects usually evolve over time with applications being adopted and abandoned as needs and technologies change, initiatives that can adapt to emerging constraints and opportunities might potentially be more successful.^20^ Overlooked in research has so far also been the possibility that eHealth could be an innovative way to embrace new models of service delivery that go beyond the adoption of specific applications. To understand these wider system changes and evaluate how engagement of providers, patients and public may lead to more distal beneficial outcomes, approaches that move past what might be termed a ‘narrow’ success of eHealth could potentially be more useful than ones related directly to clinical and technological imperatives.

As emphasised by Hübner,^21^ innovations in eHealth should be characterised not only by their novelty, but by their broader use and usefulness, adoption by patients and providers, integration into routine practice, and applicability in real-word settings. Based on this notion, and guided by frameworks on digital^22^ and organisational^23^ readiness, collaborative governance,^24^ integration,^25^ partnership^26^ and community participation,^27^ the current paper is interested in how a novel eHealth initiative in rural northern Sweden – the virtual health rooms (VHRs) – might improve access to person-centred care and strengthen community health systems. Rather than presenting the concepts guiding our analysis here, they are defined and integrated in the combined results and discussion section below to make explicit the links between theory and findings as part of an abductive-retroductive analytical process.^28^ Through this approach, our specific focus was to explore how eHealth initiatives – like the VHRs – could; (*i*) support provision of care that aligns more closely with rural residents’ opportunities to identify health-related needs as well as seek, reach and obtain subsequent services,^29^ and (*ii*) promote interactions between actors involved in “producing, advocating for, and supporting health in communities and households outside of, but existing in relationship to, formal health structures”^30^ (p. 114). We posit that these distal outcomes can be achieved at least partly irrespective of whether the narrow ambitions of the VHRs themselves are met.

###  The “Virtual Health Rooms” 

This study is situated in an area popularly known as *Norrland*, which covers about 60% of the Swedish land area, but is inhabited by only 12% of the total population. With roughly 5 residents per km^2^, this is a sparsely populated region where people live in small villages in the inland or in somewhat larger cities along the coast. The idea of VHRs emerged in 2011/2012, in response to population ageing, a lack of financial resources, a marginalisation of rural health systems, and a consequent scarcity of basic primary care services in these areas. The first VHR was opened in 2013 as a collaboration between Sweden’s Centre for Rural Medicine, Region Västerbotten (provincial health department) and Storuman municipality with the aim of providing a range of ‘self-service’ digital technologies in a customized facility that connects communities and distant providers.^31^

The first (and still operating) VHR is located in the village of Slussfors which has a relatively elderly population of about 120 people, served by a cottage and main regional hospital, almost 60 km and 300 km away, respectively. The VHR is accessed through referral from medical staff and via self-referral, and includes amenities for teleconsultations as well as self-administered blood testing and health checks (eg, blood pressure and heart rate). Test results are automatically uploaded to a database linked to patient records. It is thus possible to use the room and equipment without assistance, although experiences so far indicate that an assistant nurse, friend or family member often accompany the patients. In an initial evaluation of the VHR in Slussfors, women seemed to be more content than men with its contribution to their healthcare while being less satisfied with its technical performance.^31^

In 2017, a project commenced to establish 8 new rooms (7 in Västerbotten and 1 in Norrbotten County). The Centre for Rural Medicine has led this process by overseeing the site selections, the local adaption of the VHRs and the installation of a basic equipment package. Reflecting the project’s participatory nature, the VHRs geographical location was largely informed by the interests and feedback of stakeholders and community members, even if ultimately decided upon by a steering group. As a result of these consultations, the VHRs were placed in the very few remaining inland villages that, while not being municipal administrative centres, have retained some social services (a school or community store, for example). Overall, the VHR villages have small populations (ranging from approximately 100-1000), have for decades experienced substantial out-migration, reductions in locally provided amenities and restricted access to care since even relatively proximate care facilities are stressed by workforce shortages and financial constraints.^32,33^ These characteristics may contribute to poor community engagement in eHealth,^14^ while aspects like strong senses of identity, cohesion, safety and solidarity in rural areas^34,35^ coupled with the availability of a robust information technology infrastructure and high levels of digital literacy may facilitate involvement and uptake.

In this study, the development of the 8 new VHRs in rural northern Sweden was taken as a point of departure. By identifying and eliciting ideas, assumptions and expectations that always underlie the design and development of an intervention while formalizing them into a model and testable propositions,^36^ the specific aim was to explore how and under what circumstances eHealth initiatives – like the VHRs – are expected to improve access to person-centred care and strengthen community health systems, especially for elderly in rural areas.

## Methods

###  Study Setting and Design 

The current research is conducted in the Swedish context more generally, and northern Sweden in particular. In this setting, the heavily decentralized healthcare system has a long tradition of being publicly funded, with governance structures fragmented into 21 regions (responsible for hospital care and healthcare centres) and 290 municipalities (providing, for example, long-term care for the elderly and disabled, and school health). For quite some time, the Swedish healthcare system has been dominated by investments in hospitals and specialized care. This means that the primary care system is considered relatively weak in comparison to many other countries. Despite long-standing ambitions to expand resource allocation, ‘front-line’ services currently receive only about 15% of the regional budgets.^37^ In parallel to these constraints, a substantial reduction in the number of hospital beds during the last 20-25 years (from about 5.2 to 2.2 per 1000 inhabitants) has contributed to a shift in responsibility for elderly long-term care from regions to municipalities and communities. This shared accountability compounded by a lack of coordination between actors has resulted in a fragmented care organization with unclear mandates, and where opportunities for synergies in health promoting and curative services have been largely overlooked.^2^

In line with our explorative aim, this study was designed around the realist evaluation methodology^36,38,39^ and with thematic analysis^40^ being used to scrutinize data collected through qualitative realist interviews.^41,42^ Following an emergent design, the analysis was conducted in an abductive-retroductive way by moving back and forth between data and existing conceptual frameworks to unearth potential mechanisms and how they may be activated in certain contexts to yield intended outcomes.^28^ In particular, this research is part of a larger research project and comprises the first in a series of studies that attempts to increase our understanding about the ways through which eHealth initiatives – like the VHRs – can improve access to person-centred care and strengthen community health systems.

###  Methodological Approach 

Realist evaluation is a theory-driven research strategy with origins in the realist philosophy of science that is concerned, not only with *whether *an intervention manages to meet its objectives, but about *how *it works, *for whom*, *in what conditions *and *why.*^38^ Underlying this approach is a view of the world as an open system, where events or effects occur as a result of complex and often unobservable, yet real interactions between human agency and social structures.^43^ Central to the methodology is thus the idea that interventions are underpinned in design and functioning by explicit or implicit theories comprising assumptions about ways through which a programme ‘might cause change.’^38^ When conducting a realist evaluation, a defining initial step is to elicit these underlying *programme theories *– their constituents and interconnected elements – by mapping them, bringing them to the surface and converting them into hypotheses suitable for empirical scrutiny.^36,44^ This means that the generative mechanisms within a programme and the contextual conditions that could activate them to produce intended and unintended outcomes are first identified; a process that involve applying abductive-retroductive reasoning on data collected using ‘theory-gleaning’ approaches such as realist interviews.^28,42^ As part of the first step, the information is then conceptualized into context-mechanism-outcome (CMO) configurations and cast as an ‘if … then … because’ proposition.^39,45^ The CMO heuristic tool as initially developed by Pawson and Tilley,^39^ has later been expanded into an intervention-context-actor-mechanism-outcome (ICAMO) configuration^45^ to account for the role of programmes in offering resources to actors who interpret and/or act on them.^46^ The analytical unit of realist evaluations – the CMO or ICAMO configurations – are then tested and refined in subsequent steps to assess the extent to which theory and practice corresponds, and provide a basis for intervention improvements.^38^

By conducting the first step in the realist evaluation cycle, in this research we have identified ideas, expectations and assumptions that underlie the design and development of the VHRs, and elicited an initial programme theory that explains how and under what circumstances eHealth initiatives are expected to work. Aspects of the intervention, context, actors, mechanisms and outcomes have been combined into an ICAMO configurational model and then translated into a testable hypothesis and a structured programme theory using the ‘if … then … because’ phraseology.^45^ In line with Pawson,^36^ the mapping of pre-existing contexts in which the VHRs are embedded have followed a multilevel structure that vary from the micro to the macro while entailing a focus on stakeholder characteristics, organisational settings and wider societal structures. By referring to hidden drivers that have ‘causal tendencies’ and provide links between intervention and outcomes when activated within or between stakeholders in certain contexts, through the generative mechanisms we have strived to capture potential changes in the reasoning or behaviours of these actors.^46^

###  Participants and Data Collection 

Participants in this study included key stakeholders “whose job it is to monitor what goes on”^41^ (p. 349) and that were likely to have knowledge about how and under what circumstances the VHRs are expected to work. The selection of participants was thus purposively limited to professionals involved in the development of the VHRs and ones holding managerial positions in different sections of the region and the municipality. In total, 8 individuals (6 men and 2 women) aged 37-60 years who worked in the areas of eHealth, business development, digitalisation, service design and process management participated in the study. Using existing contacts, these individuals were contacted, informed about the study and invited to participate via e-mail.

Two authors (FJ and AKH) conducted 8 realist interviews^41,42^ between October 2019 and January 2020 using probing questions and written thematic guides related to the overall purpose of the intervention as well as how, for whom, in what conditions and why it might work. The interviews were digitally recorded, conducted in Swedish either face-to-face at the participant’s office or via telephone, transcribed verbatim and lasted between 45 and 75 minutes (with an average of 55 minutes).

###  Data Analysis and Synthesis 

The analysis started directly after each interview when the responsible researchers (FJ and AKH) independently summarised their understandings. This meant that there was an overlap in the data collection and analysis, and with participants being successively recruited based on insights gained from the initial analyses. Thematic analysis^40^ was used to analyse the data in an abductive-retroductive process.^28^ We thus oscillated back and forth between theoretical concepts and the empirical material to imagine the existence of and theorize about mechanisms through which the intervention might influence the reactions of actors in certain contexts to generate the anticipated effects.^47^ Specifically, while the analysis of interviews partly guided the inclusion of different frameworks (data-driven), after they had been integrated into the research process, the team went back to the material to further scrutinize the data using the concepts of digital^22^ and organisational^23^ readiness, collaborative governance,^24^ integration,^25^ partnership^26^ and community participation^27^ as analytical lenses (theory-driven).

Based on the 6 phases of thematic analysis^40^ coupled with abductive-retroductive theorizing,^28^ the data was analysed in the following steps. Firstly, 2 authors (FJ and AKH) read the transcripts to familiarise with the data and write down initial ideas. Secondly, the transcripts were systematically coded by the same authors with a focus on how aspects of the intervention, context, actors, mechanisms and outcomes played out across the entire data set. Thirdly, codes with similar content were collated into preliminary themes according to the ICAMO structure and organised in a matrix table. Through discussions between all authors (FJ, DBC, IG and AKH), the fourth step involved a review of the matrix table and the initial generation of a configurational model. The development of the model implied that links between the ICAMO elements were formulated by comparing possible explanations to the anticipated effects based on the idea that outcomes arise from interactions between mechanisms and contexts.^38^ The specificities of each ICAMO theme and the model was then refined with regard to existing frameworks and concepts in a fifth recursive step. Finally, all themes and the final model were described in writing to by linking data extracts that were translated from Swedish to English with deeper argumentation in relation to our aim.

###  Results and Discussion – Outlining and Combining ICAMO Elements 

In this combined result and discussion section, the developed themes are presented in relation to existing conceptual frameworks following our emergent design and abductive-retroductive analytical process. The overall findings are summarized in Figure, and in accordance with the ICAMO structure, we start by describing *contextual *factors varying from the macro to the meso and the micro, considered relevant to understand the conditions in which the *intervention *(the VHRs) are presumed to work. We then continue depicting intermediary *outcomes *that the intervention is expected to generate, thereafter outlining important collective and individual *mechanisms *that might be triggered within and between *actors *in this setting to improve access to person-centred care and strengthen community health systems.

**Figure:**
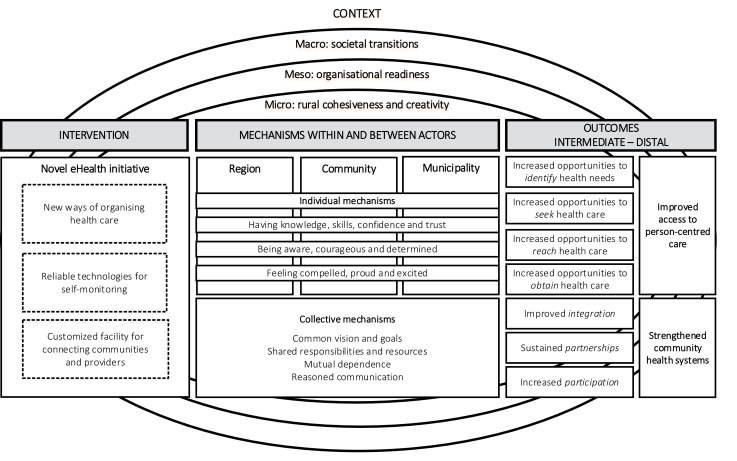
Model Combining ICAMO Elements by Specifying Intervention Components That May Contribute to Different Outcomes by Triggering Various Individual and
 Collective Mechanisms Within and Between Actors in a Certain Multilevel Context. Abbreviation: ICAMO, intervention-context-actor-mechanism-outcome.

###  Multilevel Context 

Following a classical multilevel structure,^36^ the contextual factors of presumed importance were grouped into 3 dimensions. Firstly, a macro layer which captures distal aspects and broader structural changes that may shape the need for as well as the opportunities to roll out and implement the VHRs. Secondly, a meso layer which encompasses organisational circumstances that may stretch across institutional boundaries

 to facilitate the VHR implementation. Lastly, a micro layer which concerns community characteristics believed to be of relevance for local uptake of the VHRs.

####  Macro Level: Societal Transitions 

By synthesising the participants’ narratives, the theme ‘societal transitions’ was developed to acknowledge a host of potentially important and supportive macro level influences. In line with the framework on digital readiness,^22^ jointly these illustrate how the design and implementation of eHealth initiatives should align more generally, albeit closely, with the trends, visions and road maps for change ongoing in various parts of society.

Extending across various domains, societal transitions comprised current *demographic *and *epidemiological changes *which, coupled with strained finances, could force northern Swedish regions and municipalities to develop new ways of working in order to manage their responsibilities and meet the growing needs of ageing populations. As explained, for example, by Erik (PHC manager) such transitions might require an increased technification as well as personalization of care.

*“We know we will get these older ones and that we won’t have [enough] young people to take care of them if we continue to work as we do now. This means that we need to and we will... like ‘technicalize’ the care, in a variety of ways, and find opportunities for people to manage their own health to a greater extent.”*

In parallel to these larger structural changes, broader *digital transformations *ongoing in various parts of society was seen potentially important. In this regard, the participants discussed how existing technologies, our increasingly “technology-driven behaviours” and a rising awareness of the potential benefits of technical solutions may act to facilitate the VHR implementation. However, at the same time, some also stressed that as a society we may be at the edge of, rather than having completely entered into, an era where the digital market is fully mature. Instead, we appear to be at a critical juncture in time when it comes to digitalisation where “*a lot [still] does not work, which means people are lost and don’t know ‘how will this be?*’” (Erik, PHC manager).

Another macro level circumstance portrayed as potentially important was the presence of a “*national movement*” regarding the concept of ‘good and close care’ (‘god och nära vård’) in focus of the recent healthcare reform.^3^ This emphasis could be seen in participant reports of perceived governmental endorsements for local initiatives associated with the policy. It also emerged from descriptions of how regional and municipal actors currently engage in dialogues about the practical implications of the concept by addressing questions like “what is person-centred care? [And] What does it mean?” (Anna, healthcare strategist). Adding to these aspects, the need for various *cultural shifts *was reflected, for example, by Helen (head of eHealth department) who stressed the need to find alternative ways of working, but more importantly, of challenging established professional identities; of transitioning towards a more holistic view where “the citizen or patient is the owner of his or her own life, health and care process”; and of ultimately changing attitudes and perceptions within the healthcare system. In accordance with, but as a slight nuance to the final remark, Hans (former healthcare director) talked about how mind-sets are indeed shifting, albeit only very slowly:

“*I mean, the professionals’ view of things in 1970 and 2020 are not the same, but it’s a very slow change, and it has been... what shall we say, the primary care was designed in the end of the 1960s, the beginning of the 1970s, and until now or just recently anyway, it has looked like it did then with formation of staff, ideas, everything. And also the ideology of how it should and should not be that many have like... clung onto, stating that nothing else can be relevant. But then you might ask ‘in 50 years, maybe the needs have changed somewhat, right? Is it possible that the staff formation you had then is not optimal now?’*”

####  Meso Level: Organisational Readiness 

Contextual factors that could influence the VHR implementation at meso levels by shaping the work within and spanning across regional and municipal boundaries was captured within the theme ‘organisational readiness.’ Based on the participants’ discussions and in accordance with the work of Weiner,^23^ it draw attention to conditions that could influence the administrative preparedness of actors and the existence of collaborative arrangements between them.

With regard to the former issue focusing on preparedness, emphasised was the presumed value of ensuring that remuneration models (eg, around compensation for healthcare visits); information security procedures (eg, around data handling and sharing); and technological infrastructures (eg, around computer systems) were functioning to manage an increased use of digital solutions. As reflected in the concept of ‘digital readiness,’ these aspects align with the literature stressing the importance of systems and structures, for example, around information governance and data management being “fit for purpose”^22^ (p. 10). Adding to these organisational aspects, the participants mentioned the importance of having a “fairly stable staffing” situation, “generic competencies” regarding team work and specific expertise on process management. The presence of an open workplace climate that could be somewhat tolerant to periods of ‘trial and error’ where people are allowed make, talk about and learn from mistakes as discussed by Helen (head of eHealth department) was also seen as central.

 With respect to aspects focusing on collaboration, the participants emphasised firstly the need for increased coordination and strengthened relationships between municipalities and the region, and secondly, the existence of organisational factors that could facilitate these efforts. Examples of these latter conditions included the presence of regulations and structures for sharing data and information more easily – something that could potentially move us beyond the “drainpipes and differentiated systems we have today” (Carl, chief digital officer). To reduce fragmentation and improve connections within the region (especially between primary and secondary care), recent changes where the healthcare organisation has shifted from being provided through a large number of specialised clinics to a smaller number of geographically consolidated healthcare ‘areas’ was also seen as vital.

“*There has [for quite some time] been a push for a more ‘localized’ healthcare organisation, that is, bringing together parts of the hospitals that relate more closely with primary care to get... because the needs have become so that it is important that you collaborate (...) and in that respect, it has been considered central that you are in the same organization (Hans, former healthcare director).*”

####  Micro Level: Rural Cohesiveness and Creativity 

The theme ‘rural cohesiveness and creativity’ was developed to capture characteristics of and aspects in the micro level context. In relation to an expanding body of research exploring the situation of rural communities in the global north, features included in this theme correspond at least partly with descriptions of rural areas as spatial locations for and social representations of a life shaped by strong social ties, reflexivity and resilience.^34,35,48^ However, based on the participants’ descriptions, the theme specifically captures experiences of communities located in the remote northern Swedish inland being more unified than the larger coastal cities while also generally having more ideas about what to do by looking at things more optimistically. As discussed, for example, by Hans (former healthcare director):

“*The only place where I met people who saw some kind of light in the tunnel was in rural areas (...) there existed some kind of positive outlook. The cities are the darkest and gloomiest. They see no possibilities, just problems.*”

###  Intermediary Outcomes 

 In this study, it is assumed that the implementation of a novel eHealth initiative in northern Sweden – the VHRs – could improve access to person-centred care, especially for rural elderly populations, and to a strengthening of community health systems. However, to achieve these distal or systemic benefits, various intermediate outcomes could be seen in the interviews.

####  Extended and Expanded Pathways to Care Utilisation

As reflected in the theme ‘extended and expanded pathways to care utilisation,’ the participants described a number of ways through which the VHRs might improve access to personalised solutions. Building on the framework of Levesque and colleagues,’^29^ where 5 abilities of care recipients (perceiving, seeking, reaching, paying and engaging) are seen as interacting with 5 aspects of care delivery (approachability, acceptability, availability, affordability and appropriateness), access to person-centred care is understood as the opportunity for people and communities to identify health needs as well as to seek, reach and obtain health services. In this regard, discussed by the participants was the possibility that the VHRs could allow for a certain “spontaneity” if patients could use them for routine check-ups and also more “sporadically” as needs for self-monitoring such as around lifestyle changes emerged and were identified. As explained by Carl (chief digital officer):

“*It’s like, I go there [as a patient] to take my blood pressure and I do it... on my own initiative a little bit too, so it’s not only... It [the VHR visit] doesn’t have to be so structured, and that also means that the care can become much more individualized or adapted to individual circumstances.*”

Considering that the VHRs represents a novel and locally situated eHealth initiative that should remain adapted to the diverse circumstances of rural communities and their members, the interviews illustrated how they might also create more equal opportunities for people to seek healthcare. This aspect – which implies that services should be judged by the users as appropriate to their needs^29^– was highlighted by Anna (healthcare strategist) as she explained that the care has to be close “for everyone” irrespective of functioning or impairment.

By basically comprising a “new level of care” (Carl, chief digital officer) situated in-between home care and care at health centres or hospitals, the VHRs could also remove some barriers for rural populations to reach health services by making them more available.^29^ In particular, by providing a physical space where patients can connect with providers without being forced to buy expensive digital applications or travel long distances, the participants discussed how the VHRs might bring the formal health system closer to the communities. However, as a nuance to this presumed benefit, the mantra “digitally when possible, physically if needed” was repeated in the interviews to reflect some dissonances in both preferences to and feasibility of implementing eHealth. While this indicates that the VHRs might not be a suitable approach for every patient, clinician or health problem, it suggests that they may still increase the opportunities for rural communities to obtain care and benefit from the services.^29^

####  Enhanced Integration, Partnerships and Participation 

Captured within the theme ‘enhanced integration, partnerships and participation’ are different aspects through which the VHRs might strengthen community health systems.^30^ In relation to the work of Atun and colleagues,’^25^ who suggest that structural changes need to be managed with robust and sustainable solutions that involve at least some degree of hierarchical authority, the participants stressed the importance of improving the *integration* of this ‘new care level’ into formal health systems. More specifically, they described how the VHRs have to “feel like a real [healthcare] unit (...) and not comprise like... ten prototypes”; “move from the ‘lab stage’ to standard practice”; and actually or eventually become “part of the rest of the health system.” Adding to this emphasis, voiced was the presumed benefits of regional and municipal actors coming together in sustained *partnerships* through more horizontal systems of coordination and accountability^26^ to ensure long-term success of the VHRs and an ultimate strengthening of local health systems. As expressed by Lars (digitization strategist), “this is not something we do separately in the region and municipalities, but something for us to do together by finding collaborative solutions that provide the desired effect.” The importance of engaging community members to increase their *participation* was further emphasized by Erik (PHC manager), “if they [the people] don’t recognise themselves in the work, then we will have a democracy problem. They need to feel like we are heading somewhere, to a place that is better.” In line with Kenny and colleagues,^27^ this suggest that the involvement and influence of rural residents in the VHR implementation might be critical to develop and ensure a locally responsive health system.

###  Generative Mechanisms Acting Within and Between Actors 

By referring to real, yet latent and mostly invisible drivers that may be triggered within and between actors to produce the intended outcomes, generative mechanisms were organised along different dimensions – an ‘*individual*’ one capturing changes presumed necessary to occur in people, dyads and/ or groups; and a ‘*collective*’ one where changes were seen as arising from repeated, quality interactions of these entities as part of the interplay between networks or organisations.^49,50^

####  Individual Mechanisms 

The range of potentially vital attributes that might act or be triggered more narrowly at intra- or interpersonal levels, was captured within the generic theme ‘individual mechanisms.’ Based on the participants’ descriptions, some of these mechanisms seemed to be broadly linked to the intervention as a whole whilst other appeared to connect more closely with certain components. In terms of the latter, stressed was the importance of professionals, patients and the public having *knowledge* about the customized facilities, for example, in terms of how, when and for what they may be reached or used, as well as sufficient *skills* to manage the specific technology. However, in accordance with Lennon and colleagues^22^ rather than being ends in themselves, these aspects could also influence the actors’ preparedness to engage in the implementation by generating *confidence* and trust among involved parties that eHealth will be a way to provide and receive quality care. As Anna’s (healthcare strategist) narration illustrates:

“*A: You know, those who work with these things need to have knowledge about... or have confidence in the patients as they report their own measurements... If they don’t feel confident that the numbers are correct then they won’t trust them but double check. I mean, if you work in healthcare, you don’t take chances, you don’t take risks. You ensure that you are correct and if you feel insecure or uncertain about working digitally, then you will call the patient for a physical visit anyways, I think*.

*I: Exactly*...

*A: So the people working need to know that this is, what to say…? Even though I care for patients remotely, I am certain and confident in that ‘I provide quality care.’ So it’s like... that both clinicians and patients feel like ‘this is going to work, this is quality and I feel safe. I feel safe with the care I provide and I feel safe with the care I receive.’*”

In terms of mechanisms that related more to the VHR intervention and its implementation as a whole, the need for both *awareness*, *courage *and *determination *among involved actors was described by the participants. At the general level, these aspects were considered essential for change to occur, but more specifically, they were seen as crucial to ensure that the actors could and would; (*i*) recognise what needed to be done, and (*ii*) dare to act on and stick with the identified solution(s). Building on Emerson and colleagues’s framework on collaborative governance,^24^ these mechanisms draw attention to the importance of the region, municipalities and communities not only revealing and sharing their interests and concerns, but of identifying and analysing relevant information and its implications. Related to these issues, the participants raised the value of professionals and community members feeling *compelled *to be “at the front line” (Erik, PHC manager) as well as *proud *and *excited *about what they could accomplish with the VHRs together with others.

####  Collective Mechanisms 

Building on and coupled with the *‘individual’ *mechanisms described above, the generic theme ‘collective mechanisms’ included aspects that would allow actors to reach some consensus and shared courses of action. This could, for example, involve the development of “a model” that all involved actors – from management to clinicians and patients – have the possibility to use and appreciate (Luca, eHealth specialist and medical doctor). It could also entail a “common view about the problems and about what tools to use to facilitate the change” (Lars, digitization strategist). These ideas largely correspond with the literature suggesting that interactions which go beyond mere consultations to involve a commitment among and engagement of actors, might allow them to agree upon common objectives and to pursue these visions by sharing resources and responsibilities in mutual dependence.^24-27^ However, in order to facilitate this scope, the participants discussed the importance of actors *communicating *in ways that everyone might understand while conversing across sectors or professions, and especially, with communities. As explained by Hans (former healthcare director): “I think, to envision what ‘good and close care’ might be in rural areas, the civic dialogue is essential in order to move the work forward.” In order to be fruitful, however, previous research suggest that such conversations might require a capacity for ‘reasoned communication’^24^ (p. 12) characterised, for example, by skillful advocacy, conflict resolution strategies, constructive self-assertions and a mutual respect between involved parties.^26^

###  Eliciting the Programme Theory 

In line with our realist evaluation approach,^36,38^ we have synthesised the above findings from stakeholder interviews into an initial programme theory by adopting abductive-retroductive theorizing,^28^ a process that allowed us to elucidate assumptions about how and under what circumstance an eHealth initiative like the VHRs would and/or should work. More specifically, using Mukumbang and collegues^45^ ‘if… then…because’ phraseology, we have translated the ICAMO configuration illustrated in Figure into a testable hypothesis and developed a structured initial programme theory which can be understood as follows;

“ ***IF *** *a novel eHealth initiative, which organises healthcare in new ways while providing reliable technologies in a customized facility that connects communities and providers is implemented in a context characterised by supportive societal transitions, sufficient organisational readiness and the harnessing of rural cohesiveness and creativity*

***THEN*** *it is likely to: ( i ) extend and expand the pathways through which rural residents can utilise care, (ii) improve the integration of a new care level into the formal health system, (iii) create sustained partnerships between regions and municipalities, and (iv) increase the participation of communities in processes and practices that influence their lives*

***BECAUSE*** *the involved actors have knowledge about the intervention, skills to use the equipment and confidence as well as trust in digital solutions; because they are aware about what needs to be done and determined to carry out the work while feeling compelled, proud and excited to do so; and because they can agree upon a common vision and goals while sharing responsibilities and resources in mutual dependence through reasoned communication*

***AS A*** ***RESULT*** *rural populations living in areas where locally provided services are scarce will have better access to person- centred care while the community health system in their region will become stronger.”*

As a partial response to persistent challenges facing remote rural communities in the northern Swedish inland, and as a potential step towards achieving the ambitions of ‘good and close care,’^3^ the VHRs were developed to establish certain health services in areas where they are otherwise limited. Adding to this goal, with the above programme theory we further propose that such eHealth initiatives may be a way to support a care provision that aligns more closely with the abilities, needs and wishes of rural communities while also being a means to promote and strengthen interactions between actors involved in the delivery and utilization of care. In this regard, the research incorporates a strong sense of optimism by suggesting that the VHRs can indeed be made to work – if certain mechanisms are triggered within, and supported by, a particular context.

## Concluding Remarks

To this day, a body of research has examined the ‘success’ of eHealth interventions by focusing on the clinical and/ or technical imperative of specific applications^5,10^ while a parallel line of inquiry has indicated that initiatives like the VHRs might be successful if service designers can overcome the challenges and constraints of rural areas (such as long distances, workforce shortages, demograpical changes and lack of resources).^51^ In relation to this literature, the current study is one of the first to go beyond either scope by focusing on wider system changes and more distal beneficial outcomes that may follow from the introduction and implementation of a novel eHealth initiative in rural northern Sweden. By representing the start of a research process, it has some limitations in terms of the breadth of contributions from what will ultimately be a larger group of stakeholders. It is likely (almost certain) that new perceptions will emerge, and with existing stakeholders revising their views throughout as we follow how the VHR implementation unfolds and proceeds. What this first paper has done, however, is to unpack and synthesise ideas, expectations and assumptions about how and under what circumstances the VHRs might improve access to person-centred care and strengthen community health systems.

By presenting our findings in the shape of a structured initial programme theory that specifies how eHealth initiatives – like the VHRs – could or should work, not only have we avoided simply ‘listing’ or ‘cataloguing’ potentially important elements,^45^ but been able to develop a template to guide further analytical work and a continued gradual building of theory. In this regard, the results from our study now need to be tested in selected cases and refined in subsequent phases of research to ensure descriptive trustworthiness of the findings and assess if the VHR concept works as intended and how, for whom, in what conditions and why.^38,42^

## Acknowledgements

 We wish to thank all study participants.

## Ethical issues

 Throughout the course of this research, measures have been taken to protect the rights, privacy and integrity of the participants. This means that they were told about the study, its aim, implications and that taking part was voluntary before giving their written informed consent. To ensure confidentiality, all personal identifiers were erased from the transcripts and pseudonyms have been used thought the manuscript. Ethical approval was granted by the Swedish Ethical Review Board (Dnr. 2019-01915).

## Competing interests

 Authors declare that they have no competing interests.

## Authors’ contributions

 Conceived and designed the study (AKH, DBC, and IG); acquisition of data (FJ and AKH); analysis and interpretation of data (all authors); drafting of manuscript (FJ); critical revision of the manuscript for important intellectual content (AKH, DBC, and IG); obtained funding (AKH, DBC, and IG).

## Funding

 This work was supported by the Swedish Research Council for Health, Working life and Welfare (Forte) [Grant number 2017-00183].

## Authors’ affiliations

^1^Department of Epidemiology and Global Health, Umeå University, Umeå, Sweden. ^2^Arctic Research Centre (Arcum), Umeå University, Umeå, Sweden. ^3^School of Business and Law, CQUniversity, Rockhampton, QLD, Australia.

## References

[1] World Health Organization (WHO). The World Health Report 2008-Primary Health Care (Now More Than Ever). Geneva: WHO; 2008.

[2] SOU SOU (2016). Effektiv vård - Slutbetänkande av en nationell samordnare för effektivare resursutnyttjande inom hälso- och sjukvården [Effective care - Final report of a national coordinator for more efficient use of resources in health care].

[3] SOU SOU (2019). God och nära vård – Vård i samverkan [Good and close care - Care in collaboration].

[4] Janssen R, Hettinga M, Visser S (2013). Innovation routes and evidence guidelines for eHealth Small and Medium-sized Enterprises. Int J Adv Life Sci.

[5] Granja C, Janssen W, Johansen MA (2018). Factors determining the success and failure of eHealth interventions: systematic review of the literature. J Med Internet Res.

[6] Chaudhry B, Wang J, Wu S (2006). Systematic review: impact of health information technology on quality, efficiency, and costs of medical care. Ann Intern Med.

[7] Fatehi F, Armfield NR, Dimitrijevic M, Gray LC (2014). Clinical applications of videoconferencing: a scoping review of the literature for the period 2002-2012. J Telemed Telecare.

[8] Abimbola S, Keelan S, Everett M (2019). The medium, the message and the measure: a theory-driven review on the value of telehealth as a patient-facing digital health innovation. Health Econ Rev.

[9] Ekeland AG, Bowes A, Flottorp S (2010). Effectiveness of telemedicine: a systematic review of reviews. Int J Med Inform.

[10] Shigekawa E, Fix M, Corbett G, Roby DH, Coffman J (2018). The current state of telehealth evidence: a rapid review. Health Aff (Millwood).

[11] Bujnowska-Fedak MM, Grata-Borkowska U (2015). Use of telemedicine-based care for the aging and elderly: promises and pitfalls. Smart Homecare Technol Telehealth.

[12] Peek ST, Wouters EJ, van Hoof J, Luijkx KG, Boeije HR, Vrijhoef HJ (2014). Factors influencing acceptance of technology for aging in place: a systematic review. Int J Med Inform.

[13] Bradford NK, Caffery LJ, Smith AC (2016). Telehealth services in rural and remote Australia: a systematic review of models of care and factors influencing success and sustainability. Rural Remote Health.

[14] Hage E, Roo JP, van Offenbeek MA, Boonstra A (2013). Implementation factors and their effect on e-Health service adoption in rural communities: a systematic literature review. BMC Health Serv Res.

[15] Marx W, Kelly JT, Crichton M (2018). Is telehealth effective in managing malnutrition in community-dwelling older adults? a systematic review and meta-analysis. Maturitas.

[16] Scogin F, Lichstein K, DiNapoli EA (2018). Effects of integrated telehealth-delivered cognitive-behavioral therapy for depression and insomnia in rural older adults. J Psychother Integr.

[17] Karnoe A, Kayser L (2015). How is eHealth literacy measured and what do the measurements tell us? a systematic review. An International Journal.

[18] De Rosis S, Nuti S (2018). Public strategies for improving eHealth integration and long-term sustainability in public health care systems: findings from an Italian case study. Int J Health Plann Manage.

[19] Alami H, Gagnon MP, Wootton R, Fortin JP, Zanaboni P (2017). Exploring factors associated with the uneven utilization of telemedicine in Norway: a mixed methods study. BMC Med Inform Decis Mak.

[20] Urueña A, Hidalgo A, Arenas ÁE (2016). Identifying capabilities in innovation projects: evidences from eHealth. J Bus Res.

[21] Hübner U (2015). What are complex eHealth innovations and how do you measure them? position paper. Methods Inf Med.

[22] Lennon MR, Bouamrane MM, Devlin AM (2017). Readiness for delivering digital health at scale: lessons from a longitudinal qualitative evaluation of a national digital health innovation program in the United Kingdom. J Med Internet Res.

[23] Weiner BJ (2009). A theory of organizational readiness for change. Implement Sci.

[24] Emerson K, Nabatchi T, Balogh S (2012). An integrative framework for collaborative governance. J Public Adm Res Theory.

[25] Atun R, de Jongh T, Secci F, Ohiri K, Adeyi O (2010). Integration of targeted health interventions into health systems: a conceptual framework for analysis. Health Policy Plan.

[26] Brinkerhoff JM (2002). Assessing and improving partnership relationships and outcomes: a proposed framework. Eval Program Plann.

[27] Kenny A, Hyett N, Sawtell J, Dickson-Swift V, Farmer J, O’Meara P (2013). Community participation in rural health: a scoping review. BMC Health Serv Res.

[28] Jagosh J (2020). Retroductive theorizing in Pawson and Tilley’s applied scientific realism. J Crit Realism.

[29] Levesque JF, Harris MF, Russell G (2013). Patient-centred access to health care: conceptualising access at the interface of health systems and populations. Int J Equity Health.

[30] Schneider H, Lehmann U (2016). From community health workers to community health systems: time to widen the horizon?. Health Syst Reform.

[31] Näverlo S, Carson DB, Edin-Liljegren A, Ekstedt M (2016). Patient perceptions of a Virtual Health Room installation in rural Sweden. Rural Remote Health.

[32] Carson DB, Lundmark L, Carson DA (2019). The continuing advance and retreat of rural settlement in the northern inland of Sweden. J North Stud.

[33] Carson DB, Schoo A, Berggren P (2015). The ‘rural pipeline’ and retention of rural health professionals in Europe’s northern peripheries. Health Policy.

[34] Woods M. Rural Geography: Processes, Responses and Experiences in Rural Restructuring. London: SAGE Publications; 2005.

[35] Shucksmith M (2018). Re-imagining the rural: from rural idyll to Good Countryside. J Rural Stud.

[36] Pawson R. The Science of Evaluation: A Realist Manifesto. Thousand Oaks, CA: SAGE Publications; 2013.

[37] Swedish Association of Local Authorities and Regions (SALAR). Chart for the Regions. https://skr.se/ekonomijuridikstatistik/ekonomi/sektornisiffror/diagramforregionerna.1883.html. Accessed 20 March, 2020. Published 2020.

[38] Wong G, Westhorp G, Manzano A, Greenhalgh J, Jagosh J, Greenhalgh T (2016). RAMESES II reporting standards for realist evaluations. BMC Med.

[39] Pawson R, Tilley N. Realistic Evaluation. London: SAGE Publications; 1997.

[40] Braun V, Clarke V (2006). Using thematic analysis in psychology. Qual Res Psychol.

[41] Manzano A (2016). The craft of interviewing in realist evaluation. Evaluation.

[42] Mukumbang FC, Marchal B, Van Belle S, van Wyk B (2020). Using the realist interview approach to maintain theoretical awareness in realist studies. Qual Res.

[43] Kazi MAF (2003). The contribution of realist evaluation for practice.

[44] Pawson R, Sridharan S (2009). Theory-driven evaluation of public health programmes.

[45] Mukumbang FC, Marchal B, Van Belle S, van Wyk B (2018). A realist approach to eliciting the initial programme theory of the antiretroviral treatment adherence club intervention in the Western Cape province, South Africa. BMC Med Res Methodol.

[46] The RAMESES II Project. What is a mechanism? What is a programme mechanism?: The RAMESES II Project—Resources and training materials for realist evaluation; 2017.

[47] The RAMESES II Project. Retroduction in realist evaluation: The RAMESES II Project—Resources and training materials for realist evaluation; 2017. 29072890

[48] Shucksmith M, Brown DL. Routledge International Handbook of Rural Studies. Abingdon: Routledge; 2016.

[49] Blom B, Morén S (2011). Analysis of generative mechanisms. J Crit Realism.

[50] Dalkin SM, Greenhalgh J, Jones D, Cunningham B, Lhussier M (2015). What’s in a mechanism? Development of a key concept in realist evaluation. Implement Sci.

[51] Farmer J, Munoz SA, Threlkeld G (2012). Theory in rural health. Aust J Rural Health.

